# Activation of Self-Incompatibility Signaling in Transgenic *Arabidopsis thaliana* Is Independent of AP2-Based Clathrin-Mediated Endocytosis

**DOI:** 10.1534/g3.118.200231

**Published:** 2018-05-02

**Authors:** Masaya Yamamoto, Takeshi Nishio, June B. Nasrallah

**Affiliations:** * Graduate School of Agricultural Science, Tohoku University, Sendai, Miyagi 980-0845, Japan; † Section of Plant Biology, School of Integrative Plant Science, Cornell University, Ithaca, NY 14853

**Keywords:** self-incompatibility, *S*-locus receptor kinase, endocytosis, AP2-complex, *Arabidopsis thaliana*

## Abstract

Internalization of plasma membrane (PM)-localized ligand-activated receptor kinases and their trafficking to sorting endosomes have traditionally been viewed as functioning primarily in the down-regulation of receptor signaling, but are now considered to be also essential for signaling by some receptors. A major mechanism for internalization of PM proteins is clathrin-mediated endocytosis (CME). CME is mediated by the Adaptor Protein Complex 2 (AP2), which is involved in interaction of the AP2 μ-adaptin subunit with a tyrosine-based Yxxϕ motif located in the cytoplasmic domain of the cargo protein. In this study, we investigated the role of AP2-mediated CME for signaling by the S-locus receptor kinase (SRK), a protein localized in the PM of stigma epidermal cells, which, together with its pollen coat-localized S-locus cysteine-rich (SCR) ligand, functions in the self-incompatibility (SI) response of the Brassicaceae. Using *Arabidopsis thaliana* plants that were made self-incompatible by transformation with an *A. lyrata*-derived SRK/SCR gene pair, we tested the effect on SI of site-directed mutations in each of the two Yxxϕ motifs in SRK and of a CRISPR/Cas9-induced null mutation in the AP2 μ-adaptin gene *AP2M*. Both *in vitro* SRK kinase activity and the *in planta* SI response were abolished by substitution of tyrosine in one of the two Yxxϕ motifs, but were unaffected by elimination of either the second Yxxϕ motif or *AP2M* function. Thus, AP2-mediated CME is considered to be unnecessary for SRK signaling in the SI response.

Intracellular trafficking is known to play an important role in the regulation of signaling by plasma membrane-localized ligand-activated receptor kinases. Classical models of signal transduction, in which signaling by these receptors is deemed to occur exclusively at the plasma membrane (PM), view intracellular trafficking as serving two functions. First, trafficking of newly synthesized receptor molecules from the endoplasmic reticulum through the Golgi directs the receptor to the PM, where signal perception and transduction take place. Second, internalization of receptor molecules from the PM and their sorting to endosomes serve in signal attenuation, with receptor molecules destined either for eventual degradation in vacuoles or recycling to the PM. It has become increasingly evident, however, that endosomal localization is also required for signaling by some receptor kinases (Scita and Di Flore 2010, Sorkin and von Zastrow 2009).

A major route for internalization of cell surface receptors and their transport to sorting endosomes is the clathrin-mediated endocytosis (CME) pathway. In CME, transmembrane cargo proteins are recruited into coated pits by a process mediated by the Adaptor Protein Complex 2 (AP2 complex), which consists of 4 subunits, designated α-adaptin, β-adaptin, μ-adaptin, and σ-adaptin (Collins *et al.* 2002). The μ-adaptin subunit of the AP2 complex binds to the cytoplasmic domain of cargo proteins via a tyrosine-based endocytosis signal, known as the Yxxϕ motif, in which Y = tyrosine, x = any amino acid, and ϕ=a bulky hydrophobic amino acid such as phenylalanine, isoleucine, leucine, methionine, or valine (Sorkin 2004). The molecular components of CME were first characterized in mammalian cells (McMahon and Boucrot 2011). Homologs of these components, including the 4 subunits of the AP2 complex, are conserved in *A. thaliana*, indicating that CME occurs in plants (Kim *et al.* 2013). Indeed, analysis of mutants of the single μ-adaptin-encoding gene found in the *A. thaliana* genome, designated *AP2M* (At5g46630), has demonstrated a role for this gene in CME of several plasma membrane-localized proteins, including the cellulose synthase CESA6, the auxin-efflux carrier PIN FORMED2 (PIN2), and the brassinosteroid receptor BRASSINOSTEROOID INSENSITIVE 1 (BRI1) (Bashline *et al.* 2013; Di Rubbo *et al.* 2013; Kim *et al.* 2013).

Consistent with its endocytosis by CME, the BRI1 receptor kinase localizes to the PM and to endosomes and it contains five Yxxϕ motifs (Geldner *et al.* 2007; Irani *et al.* 2012), although a role for these motifs in endocytosis has not been reported. Interestingly, Yxxϕ motifs are found in several other plant transmembrane receptor-like kinases (RLKs), a few of which have been localized to the PM and to endosomes (Geldner and Robatzek 2008). However, for the majority of these RLKs, neither the mechanism nor the possible requirement of internalization for signaling has been addressed. One of these receptors is the S-locus receptor kinase (SRK), which is a transmembrane protein expressed in the stigma epidermal cells of self-incompatible members of the Brassicaceae (Stein *et al.* 1991). The *SRK* gene and the gene for the small pollen coat-localized S-locus cysteine-rich protein (SCR) (Schopfer *et al.* 1999), which functions as the ligand for SRK (Kachroo *et al.* 2001; Takayama *et al.* 2001), are tightly linked and highly polymorphic genes that together constitute the *S*-locus haplotype (*S* haplotype) and determine specificity in the self-incompatibility (SI) response (reviewed in Nasrallah and Nasrallah 2014). SRK is only bound and activated by the SCR that is encoded in the same *S* haplotype. This allele-specific receptor-ligand interaction, which occurs only when stigma epidermal cells and pollen are derived from plants that express the same *S* haplotype, triggers a downstream signaling cascade in stigma epidermal cells that causes inhibition of pollen germination and pollen tube growth at the surface of these cells (Nasrallah and Nasrallah 2014).

In the study reported herein, we wished to determine if CME of SRK is required for the SI response. Like other recent structure-function studies of SRK (Boggs *et al.* 2009a; Yamamoto *et al.* 2014), this study was facilitated by the availability of a transgenic self-incompatible *Arabidopsis thaliana* model (Nasrallah *et al.* 2002, 2004), in which the ease of transformation allows for efficient *in planta* testing of mutated SRK proteins generated by site-directed mutagenesis. We previously showed that several *SRK/SCR* gene pairs, including the *SRKb/SCRb* gene pair derived from the *Sb* haplotype of self-incompatible *Arabidopsis lyrata*, confer an intense and developmentally stable SI response in several accessions of the normally self-fertile *A. thaliana*, including the C24 accession (Nasrallah *et al.* 2004; Boggs *et al.* 2009a,b). This transgenic *A. thaliana* platform was recently used for live-cell imaging of functional YFP-tagged SRKb proteins in stigma epidermal cells (Rea and Nasrallah 2015). As expected, the full-length SRKb-YFP receptor was observed predominantly at the plasma membrane of epidermal cells in unpollinated stigmas. Notably, this localization of the SRK at the plasma membrane and in juxtaposition to the cell wall, which make its extracellular domain accessible to its pollen-derived SCR ligand, is critical for SRK function. Indeed, loss of SI has been observed when SRK targeting to the plasma membrane is disrupted (Yamamoto *et al.* 2014; Tantikanjana and Nasrallah. 2015), and when stigmas are treated with 5M NaCl, which causes plasmolysis and retraction of the plasma membrane from the cell wall of epidermal cells (Rea and Nasrallah. 2015; Tantikanjana and Nasrallah. 2015).

In addition to its primary localization at the plasma membrane, the full-length SRKb-YFP receptor was also observed, albeit at much lower levels, in transvacuolar strands and small intracellular vesicle-like structures of unpollinated stigmas (Rea and Nasrallah 2015). Some of these vesicles might be endosomes as inferred from a previous immunolocalization study of the *Brassica oleracea* SRK3 variant (Ivanov and Gaude 2009). Interestingly, neither of the two SRK localization studies detected a redistribution of SRK in response to self-pollination. Although this result might be related to the resolving power of the visualization methods used, the possibility that SRK is internalized was suggested by internalization of an anti-SRK3 antibody that served as a mimetic for the SCR3 ligand (Ivanov and Gaude 2009). In any case, a functional connection between SRK internalization and SRK signaling in the SI response has not been established.

To address this issue, we focused on the CME pathway as a potential mechanism of SRK internalization, and more specifically on the Yxxϕ motif-AP2M interaction, which is an essential step in CME. We generated strains that express SRKb mutant proteins in which Yxxϕ motifs were destroyed by site-directed mutagenesis and we constructed strains carrying an *ap2m* null mutation generated using the CRISPR/Cas9 gene editing method. Our analysis of these mutant strains strongly suggests that CME is not required for SRK signaling in the SI response.

## Materials And Methods

### Plant materials, construction of mutant SRKb transgenes, and generation of transgenic plants

All *Arabidopsis thaliana* plants used in this study were plants of the C24 accession (obtained from the Arabidopsis Biological Resource Center at the Ohio State University, Columbus, Ohio) and were grown at 23° under continuous light.

The highly self-incompatible strain harboring the *AtS1*_pro_*:SRKb-FLAG:SRKb*term+*SCRb* transgenes [here designated *AtS1*_pro_*:SRKb-FLAG*+*SCRb*], which expresses wild-type SRKb-FLAG in stigmas and the SCRb protein in pollen, was described previously (Yamamoto *et al.* 2014). The *AtS1*_pro_*:SRKb*(*Y600A*)*-FLAG* and *AtS1*_pro_*:SRKb*(*Y693A*)*-FLAG* mutant transgenes, all including the *SRKb* terminator, were generated by recombinant PCR using the *AtS1*_pro_*:SRKb-FLAG*+*SCRb* plasmid as template and the following primers: SRKb(Y600A)F (5′-GTTGATCGCTGAGTATTTGGAGAACCTAAGCCTTGATTCT-3′) and SRKb(Y600A)R (5′-CCAAATACTCAGCGATCAACATCTTCTCGTTTTCGTCGAC-3′) for the *AtS1*_pro_*:SRKb*(*Y600A*)*-FLAG* transgene, and primers SRKb(Y693A)F (5′-GGAACTGCCGGTTACATGTCTCCAGAATACGCGATGGATG-3′) and SRKb(Y693A)R (5′-GACATGTAACCGGCAGTTCCGACCACCTTCCTCGTGTTAG-3′) for the *AtS1*_pro_*:SRKb*(*Y693A*)*-FLAG* transgene. The transgenes were inserted into the pCAMBIA1300 plant transformation plasmid (GenBank accession number AF234296) and sequenced to confirm the absence of PCR-generated errors. The transgenes were then introduced into *Agrobacterium tumefaciens* GV3101 and subsequently into C24 plants using the floral dipping method (Clough and Bent 1998). *AtS1*_pro_*:SRKb*(*Y600A*)-*FLAG* and *AtS1*_pro_*:SRKb*(*Y693A*)*-FLAG* transformants were selected on Murashige and Skoog medium (Wako, Osaka, Japan) containing 50 μg ml^-1^ hygromycin.

### Transient expression in A. thaliana leaf protoplasts and in vitro kinase assay

The *35S*_pro_*:SRKb-FLAG:SRKb*_term_ plasmid used for transient expression of SRKb-FLAG was described previously (Yamamoto *et al.* 2014). For transient expression of SRKb(Y600A)-FLAG and SRKb(Y693A)-FLAG, *35S*_pro_*:SRKb(Y600A)-FLAG:SRKb*_term_ [designated *35S*_pro_*:SRKb(Y600A)-FLAG*] and *35S*_pro_*:SRKb(Y693A)-FLAG:SRKb*_term_ [designated *35S*_pro_*:SRKb(Y693A)-FLAG*] chimeric genes were constructed by recombinant PCR using the *35S*_pro_*:SRKb-FLAG:SRKb*_term_ plasmid as template with SRKb(Y600A)F and SRKb(Y600A)R primers or SRKb(Y693A)F and SRKb(Y693A)R primers, respectively. To construct the *35S*_pro_*:SRKb(K555R):SRKb*_term_ [designated *35S*_pro_*:SRKb(K555R)-FLAG*] chimeric gene, which encodes a kinase-dead SRKb protein, a *Stu*I-*Sac*I fragment spanning the K555R mutation was isolated from the *AtS1_pro_:SRKb(K555R)* plasmid (Tantikanjana *et al.* 2009) and inserted into *Stu*I and *Sac*I digested *35S*_pro_*:SRKb-FLAG:SRKb*_term_ plasmid. Isolation of *A. thaliana* protoplasts and transient expression analysis were performed according to Yoo *et al.* (2007). The leaves of 50 3- or 4-week-old C24 plants were cut into 0.5- to 1-mm leaf sections and incubated for 6 hr in 10 ml of enzyme solution containing 1.5%[w/v] cellulase Onozuka R-10 (Yakult Pharmaceutical, Tokyo, Japan), 0.4%[w/v] macerozyme R-10 (Yakult Pharmaceutical, Tokyo, Japan), 0.4 M mannitol, 20 mM KCl, and 20 mM MES, pH 5.7. Plasmid DNA was transfected into the protoplasts by treatment with a solution containing 5 μg plasmid DNA, 40%[w/v] polyethylene glycol 4000 (Sigma-Aldrich, St. Louis, MO), 0.2 M mannitol, and 100 mM CaCl_2_. The transfected protoplasts were incubated overnight at room temperature in a solution containing 500 mM mannitol, 20 mM KCl, and 4 mM MES, pH 5.7.

The transfected cells were collected by centrifugation and resuspended in 200 μl of lysis buffer containing 20 mM sodium phosphate buffer pH 7.4, 150 mM NaCl, 1 mM EDTA pH 8.0, 10%[v/v] glycerol, 0.1%[v/v] Triton X-100, 1 mM PMSF, 1x protease inhibitor cocktail (Cat#P9599, Sigma-Aldrich, St. Louis, MO), and 1x phosphatase inhibitor cocktail (Cat#P0044, Sigma-Aldrich, St. Louis, MO). The resuspended cells were disrupted by vortexing with 0.2 g of glass beads and the mix was centrifuged to remove cell debris and glass beads. The resulting supernatant (180 μl) was transferred to a new tube, to which 300 μl of lysis buffer and 20 μl of a 50%[v/v] suspension of anti-FLAG M2 affinity gel (Sigma-Aldrich, St. Louis, MO #A2220) were added. Following incubation for 3 hr at 4°, the anti-FLAG M2 affinity gel was washed three times with 1 ml of 20 mM sodium phosphate buffer, pH 7.4, containing 150 mM NaCl, 1 mM EDTA pH 8.0, 10%[v/v] glycerol, and 0.1%[v/v] Triton X-100, followed by two washes with 1 ml of 20 mM TrisHCl pH 7.5, 150 mM NaCl, 10 mM MgCl_2_, and 10 mM MnCl_2_. The anti- FLAG M2 affinity gel was resuspended in 20 μl of kinase buffer [20 mM TrisHCl pH 7.5, 150 mM NaCl, 10 mM MgCl_2_, 10 mM MnCl_2_, 1 mM PMSF, 1x protease inhibitor cocktail, and1 × phosphatase inhibitor cocktail], 1 μl of ^32^P-γATP was added, and the mix was incubated for 30 min at room temperature. The samples were then washed twice with 1x phosphate buffered saline containing 20 mM EDTA, and the samples were eluted with 20 μl of SDS-PAGE sample buffer (Laemmli 1970) by boiling for 5 min at 95°.

For detection of phosphorylated SRKb proteins, the samples were run on SDS-PAGE gels, the gels were dried, and radioactive signals were detected using a Storm 860 Image Analyzer (Molecular Dynamics, Sunnyvale, CA). For western blot analysis, samples were subjected to SDS-PAGE followed by transfer to Immobilon-P membranes (Millipore, Billerica, MA) as described by Towbin *et al.* (1979). SRKb-FLAG proteins were detected using a 1:1,000 dilution of monoclonal anti-FLAG antibody (Sigma-Aldrich, St. Louis, MO) as primary antibody and a 1:3,000 dilution of goat anti-mouse-IgG peroxidase-labeled antibody (Sigma-Aldrich, St. Louis, MO) as secondary antibody. Immunodetection was performed using the ECL2 system (Thermo Fisher Scientific, Waltham, MA) and exposure to X-ray film.

### CRISPR/Cas9 mutagenesis of the AP2M gene

A plasmid carrying *AP2M* sgRNA and the *Cas9* gene was generated as described by Fauser *et al.* (2014). A 20-bp spacer sequence complementary to a region spanning nucleotides 62-81 in the first exon of *AP2M* was designed using the CRISPR DESIGN website (http://crispr.mit.edu). Synthetic oligonucleotides AP2M gRNA F (5′-ATTGGCACCTACCGAGATGACGTC-3′) and AP2M gRNA R (5′-AAACGACGTCATCTCGGTAGGTGC-3′) were annealed, inserted into the *Bbs*I restriction site of pEn-Chimera and subsequently introduced into pDe-CAS9 using LR Clonase II (Thermo Fisher Scientific, Waltham, MA). The resulting plasmid, designated *AP2Ms*gRNA-*Cas9*, was introduced into C24 plants as described for *SRKb* mutant constructs. Seed from *Agrobacterium*-treated plants were sown on soil and transformants were selected by spraying seedlings with BASTA (Bayer CropScience, Berlin, Germany) at a 1:1,540 dilution. The *AP2Ms*gRNA-*Cas9* gene cassette was subsequently introduced into *AP2M*[*AtS1*_pro_*:SRKb-FLAG+SCRb*] by crossing, and *ap2m*[*AtS1*_pro_*:SRKb-FLAG+SCRb*] mutant plants were identified among the second- and third-generation progenies of the cross.

To detect the CRISPR/Cas9-generated *ap2m* mutation, genomic DNA from leaf tissues was extracted using the CTAB method (Doyle and Doyle 1987). *AP2M* fragments spanning the targeted region were amplified by PCR using the AP2M geno-F (5′-GAGATCTCGTCGAACCTCAA-3′) and AP2M geno-R (5′-ACATGGAGCTGAAAATGAAG-3′) primers, and digested with *Bsa*HI (New England Biolabs, Ipswich, MA) for 1 hr at 37°. To confirm the presence of the *ap2m* mutation and demonstrate that plants were true *ap2m* homozygotes and not chimeras, undigested PCR products were cloned into the pGEM-T Easy vector (Promega, Fitchburg, WI), and the inserts of 6 different clones were sequenced using the GenomeLab DTCS Quick Start Kit (SCIEX, Framingham, MA) and a Beckman Coulter CEQ2000XL DNA sequencer (SCIEX, Framingham, MA). To confirm the absence of *AP2Ms*gRNA-*Cas9* gene cassette in *ap2m*[*AtS1*_pro_*:SRKb-FLAG+SCRb*] plants, genomic DNA from these plants, along with the *AP2Ms*gRNA-*Cas9* plasmid as positive control, were subjected to PCR using SS42 (5′-TCCCAGGATTAGAATGATTAGG-3′) and SS43 (5′-CGACTAAGGGTTTCTTATATGC-3′) primers (Fauser *et al.* 2014). The presence of *SRKb* was demonstrated by PCR using AlSRKb3200 (5′-GCGATGGATGGGATATTCTC-3′) and AlSRKb3700R (5′-GGCTCAATGGCTTTCCAA-3′) primers.

### Pollination assays

The stigmas of buds at developmental stage 13 (Smyth *et al.* 1990) were manually pollinated with pollen grains from mature flowers under a stereomicroscope. Two hours after pollination, the stigmas were fixed, stained with decolorized aniline blue, and examined by epifluorescence microscopy as described previously (Kho and Bear 1968). Each pollination assay was performed in triplicate. Pollen was derived either from wild-type C24 plants or from *AtS1*_pro_*:SRKb-FLAG+SCRb* plants. In these assays, results were scored as incompatible when fewer than five pollen tubes are observed per pollinated stigma, as partially incompatible when 6 to 29 pollen tubes are observed per pollinated stigma, and as compatible when more than 30 pollen tubes are observed per pollinated stigma. Images of pollinated stigmas were captured using an Axio Imager M1 microscope fitted with an AxioCam MRm camera or an Axioskop microscope with an AxioCam ERc 5s camera (Carl Zeiss, Oberkochen, Germany).

### Accession numbers

The accession numbers of SRK amino-acid sequences used in this study are listed in Figure S1. The accession numbers of *A. thaliana* RLKs are NP_ 19560.1 (BRI1, At4g39400), NP_175957.1 (BRL1, At1g55610), NP_ 178304.1(BRL2, At2g01950), NP_001190904.1 (BAK1, At4g33430), NP_177710.1 (CLV1, At1g75820), NP_196345.1 (EXS, At5g07280), NP_177328.1 (SERK1, At1g71830), NP_174683.1 (SERK2, At1g34210), NP_180201.1 (ERECTA, At2g26330), NP_194578.1 (HAESA, At4g28490), NP_176789.1 (TMK1, At1g66150), NP_199445.1 (FLS2, At5g46330), and NP_193869.1 (ARK3, At4g21380).

### Data availability

Strains and plasmids are available upon request. The authors affirm that all data necessary for confirming the conclusions of the article are present within the article, figures, and tables. Supplemental material available at Figshare: https://doi.org/10.25387/g3.5970091.

## Results and Discussion

### Two potential clathrin-mediated endocytosis Yxxϕ motifs are conserved in the kinase domain of SRK variants

**Figure 1:**
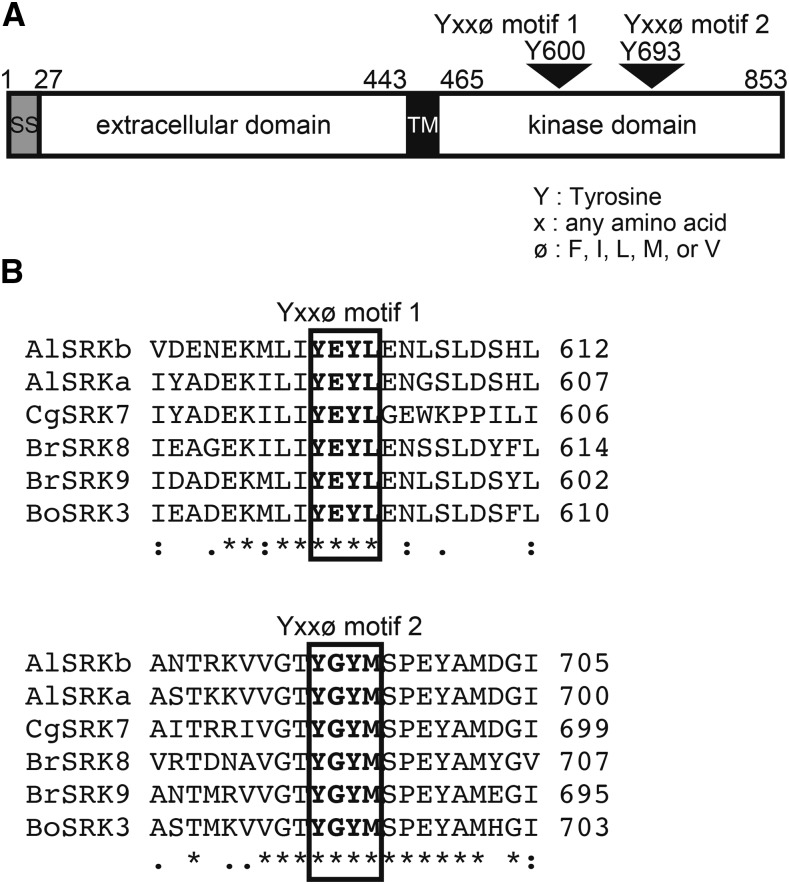
Endocytosis signals in SRK proteins. (A) Domain structure of the *Arabidopsis lyrata* SRKb protein. The positions of the two Yxxϕ motifs in the kinase domain are shown. SS: single sequence; TM: transmembrane domain. (B) Alignment of amino-acid sequences in the regions spanning Yxxϕ motif 1 (top) and Yxxϕ motif 2 (bottom) in SRKb with the corresponding regions of SRK protein variants from several Brassicaceae species, *A. lyrata* (Al), *Brassica rapa* (Br), *B. oleracea* (Bo), and *Capsella grandiflora* (Cg). Sequence alignments of all 78 SRK variants analyzed are shown in Figure S1. Asterisks, colons, and periods indicate identical amino acids, amino acids having strongly similar properties, and amino acids having weakly similar properties, respectively.

As shown in Figure 1A, the *A. lyrata* SRKb protein possesses two Yxxϕ motifs (hereafter designated Yxxϕ motif 1 and Yxxϕ motif 2) within its kinase domain, which might potentially function as endocytosis signals for CME of the receptor. To assess the possible conservation of these motifs in SRKs, the Clustal Omega program (Sievers *et al.* 2011) was used to align the kinase-domain amino-acid sequences of SRKb and SRK variants available in public databases. Seventy-eight SRK variants, including nine from *A. lyrata* (AlSRKs), 12 from *A. halleri* (AhSRKs), one from *Capsella grandiflora* (CgSRK), 25 from *Brassica rapa* (BrSRKs), and 31 from *B. oleracea* (BoSRKs) were aligned with SRKb. As shown in Figure 1B and Figure S1, Yxxϕ motif 1 [YEYL (Y600E601Y602L603) in AlSRKb] is conserved among all SRKs analyzed, while Yxxϕ motif 2 [YGYM (Y693G694Y695M696) in AlSRKb] is conserved in all SRKs except BoSRK1, in which YGYM is replaced by CGYM. In any case, the conservation of these two Yxxϕ motifs in the overwhelming majority of SRK variants in various Brassicaceae species suggests that these motifs are important for SRK trafficking or activity.

### The Y600 residue in Yxxϕ motif 1 of AlSRKb is required for kinase activity

**Figure 2:**
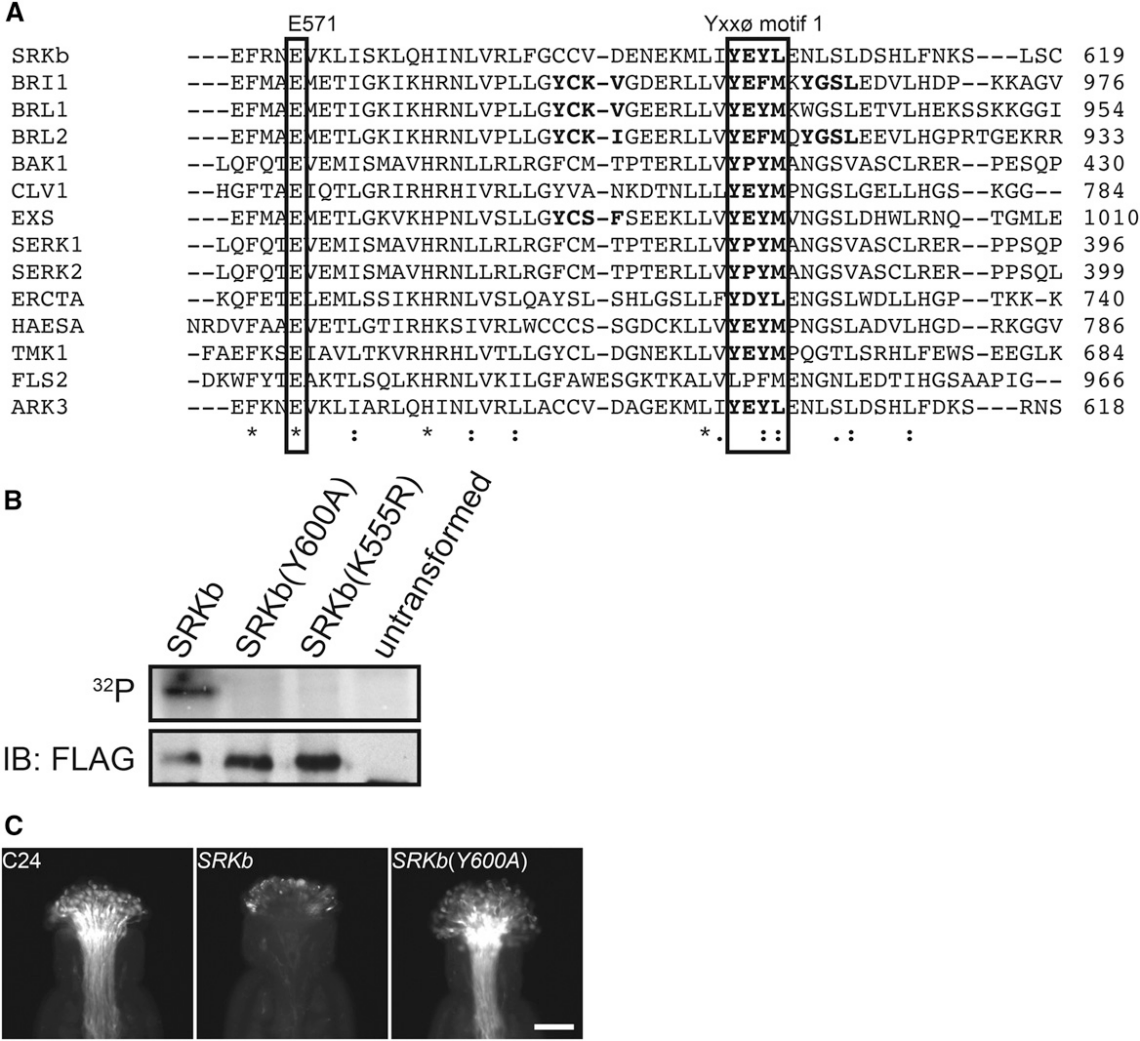
The Y600 residue in Yxxϕ motif 1 is conserved in several *A. thaliana* receptor kinases and is required for SRKb kinase activity and SI. (A) Alignment of amino-acid sequences in the region spanning Yxxϕ motif 1 in SRKb with the corresponding regions of the *A. thaliana* RLKs BRI1 (BRASSINOSTEROID INSENSITIVE 1), BRL1 (BRI1-LIKE 1), BRL2 (BRI1-LIKE 2), BAK1 (BRI1-ASSOCIATED RECEPTOR KINASE), CLV1 (CLAVATA 1), EXS (EXTRA SPOROGENOUS CELLS), SERK1 and, SERK2 (SOMATIC EMBRYOGENESIS RECEPTOR-LIKE KINASE 1 and 2), ERECTA, HAESA, TMK1 (TRANSMEMBRANE KINASE 1), FLS2 (FLAGELLIN-SENSITIVE 2) and ARK3 (ARABIDOPSIS RECEPTOR KINASE 3). The location of the glutamic acid residue (E571 in SRKb) that is part of the ATP-binding site is indicated. Note that all of the receptor kinases shown, except FLS2, contain a Yxxϕ motif in the same position as Yxxϕ motif 1 in SRKb. Asterisks, colons, and periods are as in Figure 1B. (B) *In vitro* kinase assays. The figure shows SDS-PAGE electrophoretic patterns of immunoprecipitated protoplast-expressed and FLAG-tagged wild-type SRKb, the SRKb(Y600A) mutant, and an SRKb(K555R) kinase-dead mutant. Proteins from untransformed plant cells [untransformed] were used as negative control. The radioactively-labeled proteins were detected with a phosphorimager (^32^P panel), and the same samples subjected to immunoblot analysis with anti-FLAG antibody (IB:FLAG panel) were used loading controls. Note that the SRKb(Y600A) mutant protein lacks kinase activity similar to the kinase-dead SRKb(K555R) protein. (C) Pollination phenotypes of untransformed plants (C24) and transformants expressing wild-type SRKb or SRKb(Y600A) proteins. Stigmas were pollinated with SCRb-expressing pollen. Note that SRKb-expressing stigmas exhibited a robust SI response, manifested by inhibition of SCRb-expressing pollen germination and tube elongation. By contrast, SRKb(Y600A)-expressing stigmas exhibited a highly compatible response manifested by the development of numerous SCRb pollen tubes, similar to the stigmas of untransformed plants. Bar = 100 μm.

To gain some clues regarding the potential role of Yxxϕ motifs in SRK, we assessed their conservation in several *A. thaliana* RLKs. Figure 2A shows an alignment of the amino-acid sequences of the region that spans Yxxϕ motif 1 in SRKb with the corresponding regions of several Yxxϕ-containing *A. thaliana* RLKs as well as the FLS2 receptor, which contains no Yxxϕ motifs (Geldner and Robatzek 2008). All RLKs included in the alignment, with the exception of FLS2, contain a Yxxϕ motif at a location equivalent to that of Yxxϕ motif 1 in SRKb (Figure 2A).

In BRI1, this conserved Yxxϕ motif is YEFM, and the role of its tyrosine residue (Y956), which corresponds to Y600 in SRKb (Figure 2A), has been elucidated. Structural analysis of BRI1 has shown that Y956 contributes to the formation of the ATP binding site in the BRI1 kinase domain by forming a hydrogen bond with the glutamic acid residue E927 (Bojar *et al.* 2014), which corresponds to E571 in SRKb (Figure 2A and Figure S1). Additionally, biochemical analysis has shown that a mutation of the Y956 residue in BRI1 eliminates kinase activity (Oh *et al.* 2009). To determine if the Y600 residue of SRKb is also important for kinase activity, possibly by contributing to ATP-binding site formation, we analyzed the activity of site-directed SRKb mutants that were transiently expressed under control of the CaMV *35S* promoter in *A. thaliana* protoplast cells prepared from rosette leaves. Three SRK proteins carrying a C-terminal 3xFLAG epitope were analyzed: (1) wild-type SRKb-FLAG; (2) SRKb(Y600A)-FLAG, a mutant in which the Yxxϕ motif was destroyed by replacing theY600 residue with alanine; and (3) a kinase-dead SRKb(K555E)-FLAG protein, in which a glutamic acid residue replaces a lysine residue located in the putative ATP binding site that was previously shown to be required for SRK kinase activity *in vitro* (Goring and Rothstein 1992; Stein and Nasrallah 1993) and SI function *in planta* (Tantikanjana *et al.* 2009). The protoplast-expressed wild-type and mutant SRKb proteins were immunoprecipitated using anti-FLAG antibodies and incubated with ^32^P-γATP to analyze *in vitro* autophosphorylation activity. As shown by the protein blots in Figure 2B, an autophosphorylation signal was detected in wild-type SRKb-FLAG but not in kinase-dead SRKb(K555E) (Figure 2B, ^32^P panel), as expected. Additionally, no autophosphorylation signal was observed in SRKb(Y600A)-FLAG (Figure 2B, ^32^P panel), despite the fact that this mutant protein was synthesized and precipitated at levels equivalent to those of wild-type SRKb-FLAG (Figure 2B, FLAG panel). These results demonstrate that the Y600 residue is required for SRKb kinase activity.

To confirm the expectation that the kinase defect in SRKb(Y600A) affects the ability of the mutant receptor to activate the SI response *in planta*, an *AtS1*_pro_*:SRKb*(*Y600A*)*-FLAG* chimeric gene was constructed, in which the promoter of the *AtS1* gene, which is highly active specifically in stigma epidermal cells (Dwyer *et al.* 1994), drives expression of the SRKb(Y600A)-FLAG mutant protein. This gene was introduced into *A. thaliana* C24 plants, and a total of 15 transformants were generated. Stigmas of all *AtS1*_pro_*:SRKb*(*Y600A*)*-FLAG* transformants were pollinated with SCRb-expressing pollen (hereafter SCRb pollen) derived from *AtS1*_pro_*:SRKb-FLAG+SCRb* plants, which express wild-type SRKb-FLAG in stigmas and the SCRb protein in pollen (Yamamoto *et al.* 2014). In contrast to the strong inhibition of SCRb pollen exhibited by stigmas expressing wild-type SRKb-FLAG, large numbers of elongated pollen tubes were observed in the stigmas of all *AtS1*_pro_*:SRKb*(*Y600A*)*-FLAG* transformants, similar to untransformed C24 plants (Figure 2C). Thus, the Y600 residue was revealed to be essential for both SRKb kinase activity and its function in SI.

### Elimination of Yxxϕ motif 2 does not disrupt SRKb kinase activity and function

**Figure 3:**
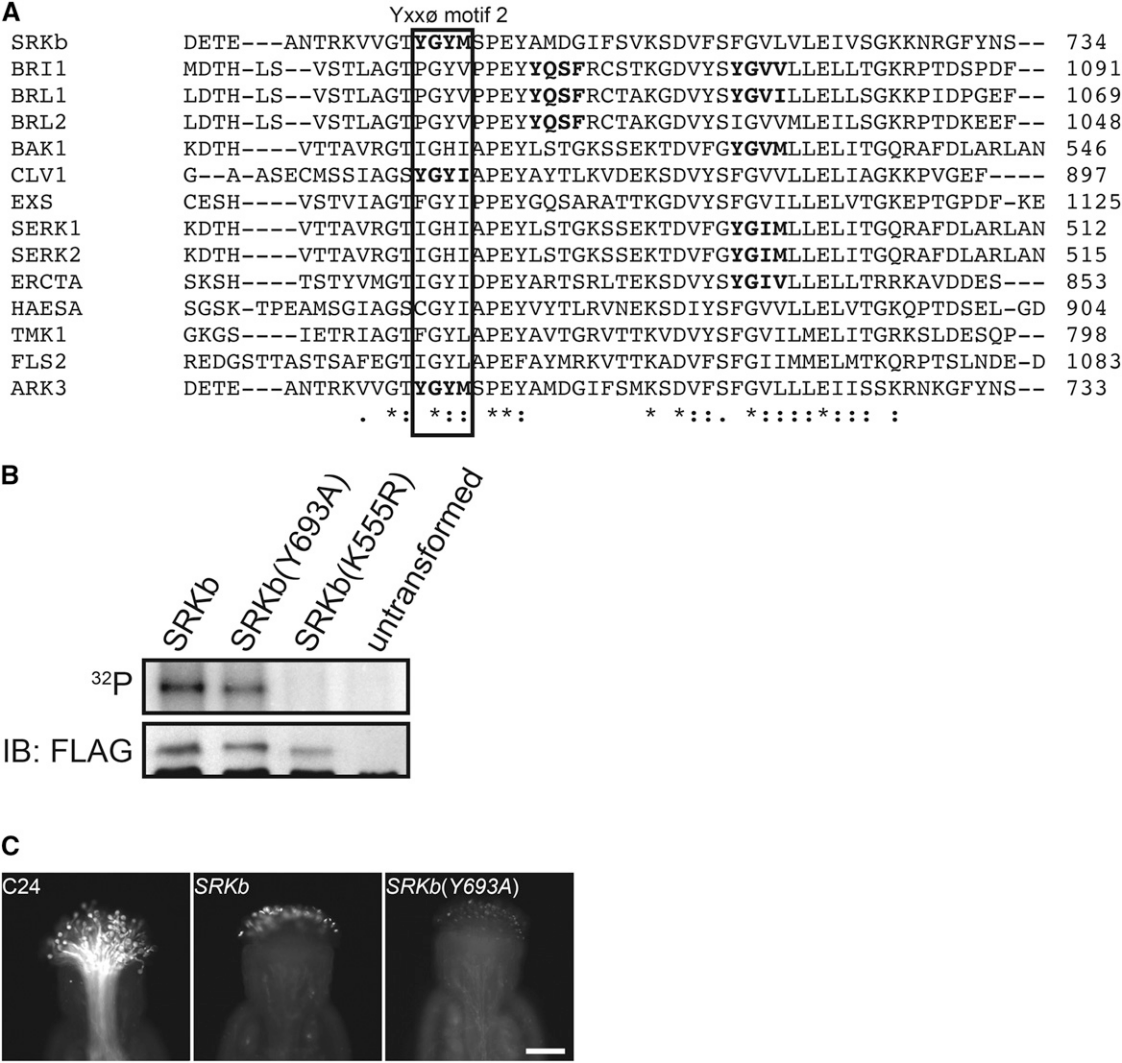
The Y693 residue of Yxxϕ motif 2 is not conserved in *A. thaliana* receptor kinases and is not essential for SRKb kinase activity and SI. (A) Alignment of amino-acid sequences in the region spanning Yxxϕ motif 2 in SRKb with the corresponding regions of several *A. thaliana* RLKs. Except for CLV1, the SRKb Yxxϕ motif 2 is not conserved in the *A. thaliana* proteins. Asterisks, colons, and periods are as in Figure 1B. (B) *In vitro* kinase assay of wild-type SRKb, the SRKb(Y693A) mutant, and the SRKb(K555R) kinase-dead protein. Protoplast-expressed proteins were prepared and assayed by autoradioagraphy (^32^P panel) or immunoblot analysis (IB:FLAG panel) as described in Figure 2B. Note that SRKb(Y693A) exhibits kinase activity similar to wild-type SRKb. (C) Pollination phenotypes of untransformed plants (C24) and transformants expressing wild-type SRKb or SRKb(Y693A) proteins. Stigmas were pollinated with SCRb-expressing pollen. Note that the stigmas expressing SRKb(Y693A) exhibit a robust SI response similar to the stigmas expressing wild-type SRKb. Bar = 100 μm.

Unlike the Y600 residue, the Y693 residue in Yxxϕ motif 2, which is located in the activation loop of the SRKb kinase domain is not conserved among *A. thaliana* RLKs (Figure 3A). However, it has been shown that phosphorylation of amino-acid residues within the activation loop of BRI1 kinases enhances both the formation of the substrate binding site and catalytic activity (Bojar *et al.* 2014). We therefore examined the possibility that the Y693 residue is required for SRKb kinase activity and function. Using the transient expression system and *in vitro* kinase assay described above, an SRKb(Y693A)-FLAG mutant protein was analyzed along with wild-type SRKb-FLAG and kinase-dead SRKb(K555R) proteins. As shown in Figure 3B, SRKb(Y693A) protein exhibited an autophospholylation signal equivalent to that observed for wild-type SRKb-FLAG. This result indicates that the Y693A mutation does not affect the phosphorylation activity of SRKb, at least in its ligand-unbound state.

To assess the function of the SRKb(Y693A)-FLAG *in planta*, C24 plants were transformed with the *AtS1*_pro_*:SRKb*(*Y693A*)-*FLAG* chimeric gene and 11 independent transformants were generated. In pollination assays with SCRb pollen, the stigmas of five *AtS1*_pro_*:SRKb*(*Y693A*)-*FLAG* transformants were found to exhibit an incompatibility phenotype that was as intense as that exhibited by stigmas expressing wild-type SRKb-FLAG (Figure 3C). Thus, the Y693 residue and Yxxϕ motif 2 are not required for SRKb function and SI *in planta*.

### A CRISPR/Cas9-induced null mutation in AP2M does not affect the SI response of SRKb-expressing stigmas

**Figure 4:**
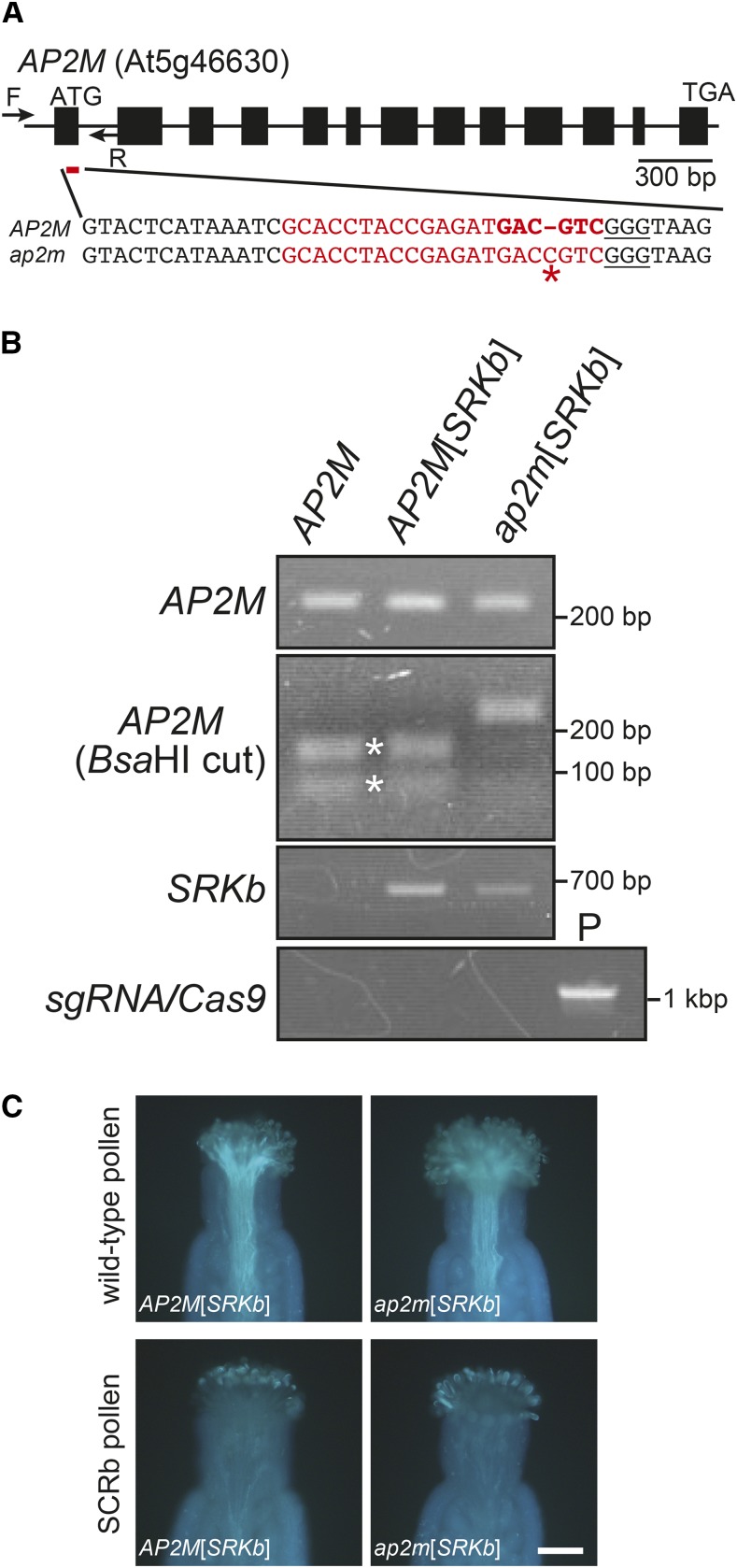
A null *ap2m* mutation does not disrupt SRKb function and SI. (A) Structure of the *AP2M* gene and sequence of the *CRISPR/Cas9*-targeted site. The *AP2M* gene structure is depicted with exons representing boxes and lines representing introns. Arrows represent annealing sites of the forward [F] and reverse [R] primers used for *AP2M* genotyping (Figure 4B) and the red line below the first exon shows the location of the targeted site. In the nucleotide sequences of the region containing the targeted site in wild-type (*AP2M*) and mutant (*ap2m*), the sequence of the sgRNA spacer is shown in red characters, the Protospacer Associated Motif (PAM) sequence GGG is underlined, and the *Bsa*HI restriction site is highlighted in bold red characters. The asterisk shows the cytosine insertion in *ap2m* that abolishes the *BsaH1* restriction site. (B) Genotyping of *AP2M*, *SRKb*, and the CRISPR/Cas9 gene cassette in C24 untransformed wild-type C24 (*AP2M*), *AP2M*[*AtS1*_pro_*:SRKb-FLAG+SCRb*] (*AP2M*[*SRKb*]), and *ap2m*[*AtS1*_pro_*:SRKb-FLAG+SCRb*] (*ap2m*[*SRKb*]) plants. The *AP2M* PCR products (top panel) were digested with *Bsa*HI enzymes [*AP2M* (*Bsa*HI cut) panel]. The 162-bp and 81-bp digestion products are indicated by asterisks. Note that the *AP2M* fragments amplified from *ap2m*[*AtS1*_pro_ *AtS1*_pro_*:SRKb-FLAG+SCRb*] DNA were not digested with *Bsa*HI. The presence of the *AtS1*_pro_*:SRKb-FLAG+SCRb* transgenes was assessed by PCR with *SRKb*-specific primers (*SRKb* panel), and the *AP2MsgRNA-Cas9* gene cassette was detected by PCR using DNA of the *AP2MsgRNA-Cas9* transformation plasmid as positive control (“P” lane in the *sgRNA-Cas9* panel). (C) Pollination phenotypes of *ap2m*[*AtS1*_pro_*:SRKb-FLAG+SCRb]* (*ap2m*[*SRKb*]) mutant plants and *AP2M*[*AtS1*_pro_*:SRKb-FLAG+SCRb*] (*AP2M*[*SRKb*]) control plants. The images show the pollination responses toward pollen from untransformed wild-type C24 plants (upper panel) and SCRb pollen (lower panel). The genotype of stigmas used for pollination is indicated in each panel. Note that the *ap2m*[*AtS1*_pro_*:SRKb-FLAG+SCRb*] stigma shows a compatible response toward wild-type pollen and an incompatible response toward SCRb pollen. Bar = 100 μm.

Because the Y600 residue of Yxxϕ motif 1 was found to be essential for SRKb kinase activity, it was not possible to derive any conclusion regarding the role of CME in SRKb signaling. To address this issue, we focused on *AP2M*, the gene that encodes the Yxxϕ motif-binding μ-adaptin of the AP2 complex (Traub 2009) and that has been shown to be essential for CME of plasma membrane-localized proteins in *A. thaliana* (Bashline *et al.* 2013; Di Rubbo *et al.* 2013; Kim *et al.* 2013; Yamaoka *et al.* 2013). Since a panel of T-DNA insertion lines in the *A. thaliana* C24 accession is not publicly available, we used the CRISPR/Cas9 gene-editing method to generate an *ap2m* loss-of-function mutation in C24[*AtS1*_pro_*:SRKb-FLAG+SCRb*] plants. A single-guide RNA (sgRNA) was designed to introduce a mutation in the first exon of *AP2M* (Figure 4A). A plant transformation plasmid carrying the designed sgRNA along with the *Cas9* gene was generated essentially as described by Fauser *et al.* (2014). Because C24[*AtS1*_pro_*:SRKb-FLAG+SCRb*] plants are highly self-incompatible and set no seed (Yamamoto *et al.* 2014), the standard floral dipping method of transformation cannot be used with these plants. Consequently, the *AP2MsgRNA-CAS9* plasmid was first introduced into untransformed wild-type C24 plants, and the resulting transformants were crossed with a wild-type *AP2M*[*AtS1*_pro_*:SRKb-FLAG+SCRb*] plant to generate *ap2m*[*AtS1*_pro_*:SRKb-FLAG+SCRb*] homozygous plants.

Mutagenized sgRNA target sites were detected by loss of a *Bsa*HI restriction site within the targeted region (Figure 4A) using cleaved amplified polymorphic sequence (CAPS) analysis. Genomic DNA was isolated from untransformed C24, *AP2M*[*AtS1*_pro_*:SRKb-FLAG+SCRb*], and *ap2m*[*AtS1*_pro_*:SRKb-FLAG+SCRb*] plants, and 243-bp *AP2M* fragments spanning the *BsaH1* site were amplified and digested with *Bsa*HI. Figure 4B shows that digestion products of 81 bp and 162 bp were detected in untransformed and *AP2M*[*AtS1*_pro_*:SRKb-FLAG+SCRb*] plants (Figure 4B), as expected. By contrast, the amplification products of *ap2m*[*AtS1*_pro_*:SRKb-FLAG+SCRb*] DNA were not digested by *Bsa*HI (Figure 4B), confirming loss of the *Bsa*HI recognition site. Furthermore, Sanger sequencing of amplification products derived from the *ap2m*[*AtS1*_pro_*:SRKb-FLAG+SCRb*] mutant revealed the presence of a frame-shifting cytosine insertion after the 78^th^ nucleotide of the first exon of *AP2M* (Figure 4A). Moreover, amplification of *ap2m*[*AtS1*_pro_*:SRKb-FLAG+SCRb*] DNA with *SRKb*-specific primers confirmed the presence of the *AtS1*_pro_*:SRKb-FLAG+SCRb* transgenes and amplification with *Cas9*-specific primers demonstrated the absence of the *AP2MsgRNA-CAS9* gene cassette (Figure 4B).

The phenotypic consequences of the null *ap2m* mutation were investigated by manual pollination of *ap2m*[*AtS1*_pro_*:SRKb-FLAG+SCRb*] stigmas with pollen from untransformed C24 plants or with pollen from an *AP2M*[*AtS1*_pro_*:SRKb-FLAG+SCRb*] plant, followed by microscopic monitoring of pollen tube growth. When the stigmas of *ap2m*[*AtS1*_pro_*:SRKb-FLAG+SCRb*] plants were pollinated with pollen from untransformed plants (cross-pollination), numerous pollen tubes were observed (Figure 4C), indicating that the *ap2m* mutation did not disrupt stigma function. By contrast, when *ap2m*[*AtS1*_pro_*:SRKb-FLAG+SCRb*] stigmas were pollinated with SCRb-expressing pollen (self-pollination), an intense SI response identical to that of self-pollinated *AP2M*[*AtS1*_pro_*:SRKb-FLAG+SCRb*] stigmas was observed (Figure 4C). This result indicates that AP2M is not required for the SI response in the transgenic *A. thaliana* model.

### Conclusions

Our attempt to investigate the requirement of CME for SRK signaling in the SI response by eliminating each of the Yxxϕ motifs in AlSRKb produced mixed results. On the one hand, the observation that the SI response remained intact when Yxxϕ motif 2 was eliminated clearly demonstrated that this motif is not required for SRK signaling. On the other hand, elimination of Yxxϕ motif 1 caused by introducing the Y600A mutation resulted in loss of the SI response. This finding cannot be directly related to a defect in CME-mediated receptor trafficking because of the requirement of the Y600 residue for AlSRKb kinase activity. However, the fact that *ap2m*[*AtS1*_pro_*:SRKb-FLAG+SCRb*] plants, which harbored a CRISPR/Cas9-induced null mutation in *AP2M*, retained the ability to reject SCRb-expressing pollen clearly demonstrates that the *AP2M*-mediated endocytosis pathway is not required for SRKb signaling and for the ability of *A. thaliana* SRKb-expressing stigmas to mount a robust SI response. This conclusion is consistent with available evidence showing that the SRK receptor signals at the PM.

Our results do not exclude the possibility that SRK is internalized via an alternative clathrin-independent endocytosis (CIE) pathway (Doherty and McMahon 2009; Mayor *et al.* 2014). A detailed analysis of *A. thaliana* endocytic pathways concluded that under normal conditions, transmembrane proteins are internalized by CME, while lipid-anchored proteins and lipids are internalized by CIE (Baral *et al.* 2015). However, CIE has been recently implicated in internalization of BRI1, particularly in its ligand-bound form (Jaillais and Vert 2016; Wang *et al.* 2015). CIE might similarly function in SRK internalization, possibly as a post-signaling mechanism for attenuating the SI response and desensitizing the stigma epidermal cell to the SCR ligand. Further studies are required to resolve this issue. It should be noted, however, that the SI response differs from other well-studied plant receptor/ligand-mediated processes, such as BRI1 signaling, in which signal termination is essential for desensitizing the cell in readiness for further response to ligand. Rather than being a cell-wide response to ligand, SRK activation and signaling are restricted to the site of contact between a stigma epidermal cell and a “self” pollen grain. Moreover, once on the stigma surface, a pollen grain is not typically dislodged by newly arriving pollen grains (Rea and Nasrallah 2015). These features suggest that internalization for signal attenuation may not play a role in the regulation of SRK signaling.
